# Insight into the population dynamics of pathogenic bacteria causing grapevine crown gall in snowfall areas: snow cover protects the proliferation of pathogenic bacteria

**DOI:** 10.3389/fpls.2023.1198710

**Published:** 2023-06-12

**Authors:** Akira Kawaguchi, Manabu Nemoto, Sunao Ochi, Yosuke Matsushita, Tomoyuki Sato, Teruo Sone

**Affiliations:** ^1^ Western Region Agricultural Research Center (WARC) (Kinki, Chugoku and Shikoku Regions), National Agriculture and Food Research Organization (NARO), Fukuyama, Japan; ^2^ Hokkaido Agricultural Research Center (HARC), National Agriculture and Food Research Organization (NARO), Sapporo, Japan; ^3^ Institute of Plant Protection, National Agriculture and Food Research Organization (NIPP), Tsukuba, Japan; ^4^ Research Faculty of Agriculture, Hokkaido University, Sapporo, Japan

**Keywords:** grapevine crown gall, under the snow, Hierarchical Bayesian Model, Bayesian changepoint detection, vineyards and plant-insect relationship

## Abstract

Grapevine crown gall (GCG) is a significant bacterial disease caused by tumorigenic *Allorhizobium vitis* (TAV) and is prevalent worldwide. TAV infects grapevines through wounds such as freezing injuries. Although grapevines typically avoid being wounded under snow cover, GCG occurs in many commercial vineyards in snowy regions. This study investigated the TAV population in GCG gall tissues, grapevine skins, and snow on grapevine skins from six infected vineyards located in Hokkaido, Japan, an area known for heavy snowfall. TAV was isolated not only from gall tissues but also from skins and snow on skins throughout the year. Hierarchical Bayesian model (HBM) analysis revealed that the number of TAV cells in gall tissues was affected by cultivar and low temperature, while those in skins were affected by location and low temperature. Additionally, Bayesian changepoint detection (BCD) showed that the number of TAV cells in gall and skin tissues increased during winter, including the snowfall season. Furthermore, the TAV population in grapevine skins under the snow was significantly higher than those above the snow, indicating that TAV under the snow is protected by the snow and can survive well during the snowfall season. This study highlights the ability of TAV to overwinter on/in galls and skins under the snow and act as inoculum for the next season.

## Introduction

*Allorhizobium vitis* (AV) [syn. *Rhizobium vitis*, *Agrobacterium vitis*] (Mousavi et al., 2015) is a soil-dwelling bacterium, and grapevine (*Vitis vinifera* L.) crown gall (GCG) is mainly caused by tumorigenic *A. vitis* (TAV). TAV infects grapevines in many vineyards and grape-growing regions through wounds such as freezing injuries, mechanical damage, and/or grafting (Burr et al., 1998; Kawaguchi et al., 2017; Kawaguchi et al., 2021). GCG outbreaks have occurred worldwide (Burr et al., 1998). Galls commonly form on the cordons and trunks of mature and young grapevines, including one-year-old seedlings (Burr et al., 1998; Kawaguchi et al., 2017). Infected grapevines often show inferior growth, and in some cases, GCG causes partial or complete grapevine death (Kawaguchi et al., 2017).

The lack of effective GCG control methods is a serious problem. *R. rhizogenes* strain K84 inhibits gall formation caused by tumorigenic *R. rhizogenes* (New and Kerr, 1972; Kerr and Htay, 1974; Kerr, 1980; Kerr and Bullard, 2020), but it is not effective against GCG caused by TAV (Burr et al., 1998; Kawaguchi et al., 2007; Kawaguchi and Inoue, 2012). Previously, we reported that nonpathogenic AV strains VAR03-1 and ARK-1 reduced gall occurrence not only in grapevines but also in several other plant species (Kawaguchi et al., 2007; Kawaguchi et al., 2008; Kawaguchi, 2009; Kawaguchi and Inoue, 2012; Kawaguchi et al., 2012; Kawaguchi, 2013; Kawaguchi, 2014; Kawaguchi et al., 2015; Kawaguchi et al., 2017; Saito et al., 2018; Noutoshi et al., 2020; Wong et al., 2021; Kawaguchi, 2022c; Kawaguchi et al., 2023). Strain ARK-1, in particular, exhibited strong activity in controlling GCG in many vineyards and has some unique biocontrol mechanisms. ARK-1 slowed the population growth of TAV strains at inoculation sites (Kawaguchi, 2014; Kawaguchi, 2015), migrated inside plants (Kawaguchi and Noutoshi, 2022a), suppressed the expression of virulence (*vir*) genes (Kawaguchi, 2015; Kawaguchi et al., 2019), and primed the induction of certain defense genes of grapevine (Kawaguchi and Noutoshi, 2022b).

Currently, a new biopesticide made from ARK-1 is being developed and has shown positive results in field trials, effectively controlling crown gall in grapevine and other plant species. This new biopesticide is strongly required as GCG often occurs in Japan, particularly in Hokkaido (Kawaguchi et al., 2017; Kawaguchi et al., 2021). Hokkaido is one of the world’s heaviest snowfall areas, with an average snowfall of 597 cm in Sapporo, the capital of Hokkaido, over the past 30 years according to the Japanese Meteorological Agency (JMA, http://www.jma.go.jp/jma/indexe.html). In many vineyards in Hokkaido, snow covers the whole grapevines every winter, protecting them from freezing injuries that often induce GCG in grapevines under approximately -20 °C air conditions (Burr et al., 1998; Kawaguchi et al., 2017; Kawaguchi et al., 2021). Furthermore, grapevines under the snow (approximately 0°C) can avoid freezing injuries in snow-covered areas because the snow blocks out the extremely cold temperatures (Jitsuyama et al., 2022). However, despite the absence of freezing injuries in snow-covered areas, an outbreak of GCG has been observed in Hokkaido (Kawaguchi et al., 2021; Kawaguchi and Nanaumi, 2022).

Therefore, the objective of this study is to investigate the reasons behind GCG occurrence in snow-covered areas. We investigated the population dynamics of TAV in some infected vineyards in Hokkaido over two years and analyzed the factors promoting GCG occurrence.

## Materials and methods

### Population measurement of TAV in gall tissues, grapevine skins, and snow

To monitor the population dynamics of TAV, grapevine plants exhibiting gall symptoms were selected from six different vineyards located in Hokkaido, Japan (vineyard A: approximately 10-year-old *Vitis vinifera* cv. Zweigeltrebe in Yoichi, vineyard B: approximately 10-year-old V. vinifera cv. Kerner in Yoichi, vineyard C: approximately 8-year-old *V. vinifera* cv. Zweigeltrebe in Urausu, vineyard D: approximately 8-year-old *V. vinifera* cv. Kerner in Urausu, vineyard E: approximately 6-year-old *V. vinifera* cv. Auxerrois, vineyard F: approximately 10-year-old *V. vinifera* cv. Zweigeltrebe in Furano), where snow depth reaches over 1.5 meters during winter from December to March. All plants were completely covered in snow during this period, while in the Urausu vineyards, this continued until April. First, five grapevine plants were randomly selected and labeled in each vineyard, and the same plants were monitored throughout the study period from 2021 to 2023, with samplings conducted 23 times (once a month) in vineyards A and B, 11 times in vineyards C and D, 12 times in vineyard E, and three times in vineyard F. Gall tissues (ca. 1.0 g fresh weight per plant, 1 sample per plant), skins (ca. 1.0 g fresh weight per plant, 1 sample per plant), and snow covering on skins (ca. 50 mL per plant, 1 sample per plant) were collected from each of the five plants (i.e., *n* = 5). In vineyard E, galls, skins, and snow samples were collected from each of the three plants (1 to 3 samples per plant, i.e., *n* = 5) due to the small number of grapevines. Gall tissues and skins were scrubbed by hand under running tap water for 10 s, and water drops were wiped off with paper towels. To assess the TAV strains living on/in plant tissues, strains were isolated using AV selective medium, rather than quantitative PCR, and some isolates were used to verify the genetic diversity and molecular epidemic analysis in a previous report (Kawaguchi et al., 2021). Although copper is known to induce the viable-but-nonculturable condition (VBNC) in certain species of *Agrobacterium/Rhizobium* (Alexander et al., 1999), which are closely related to *Allorhizobium*, the grapevines sampled in this experiment were not exposed to any fungicides containing copper compounds. Therefore, it is unlikely that the observed TAV was in the VBNC state under the experimental conditions.

The samples were crushed in 0.9 mL of sterile distilled water using an autoclaved mortar and pestle, while snow samples were crushed without prior distillation. Ten-fold serial dilutions (total of 100 μL) of each sample were prepared and spread onto AV selective Roy and Sasser medium plates (Roy and Sasser, 1983). The plates were incubated at 25°C for 5 days, and the colony-forming units (CFU) were counted. The detection limit of this procedure was 10^2^ CFU (2.0 log_10_ CFU)/g of plant tissue or snow. Some colonies generated on the Roy and Sasser medium were confirmed by multiplex PCR, using the two TAV-specific primer sets Ab3-F3/Ab3-R4 and VCF3/VCR3, as previously described (Kawaguchi et al., 2005). As negative controls, skins and snow covering on skins were collected from five healthy grapevines (i.e., *n* = 5), and TAV strain isolation was conducted. All statistical analyses were performed using the RStudio user interface (version 1.2.5001) for R software (version 3.6.1, R Foundation for Statistical Computing, http://www.r-project.org/)”.

### Hierarchical Bayesian model

To investigate the factors influencing the number of CFU of TAV in grapevine gall tissues and skins, we performed a regression analysis using a hierarchical Bayesian model (HBM), taking into account information on cultivars, locations, and sampling-month histories obtained from various vineyards. The experimental methods used in this study followed those described in previous reports (Kawaguchi, 2020; Kawaguchi et al., 2021; Kawaguchi and Nanaumi, 2022). Weather data from vineyards A and B were collected from a weather station in Yoichi, those from vineyards C and D from Takikawa, those from vineyard E from Furano, and those from vineyard F from Sapporo, with the chosen station located no more than 20 km from each vineyard investigated. Data collection was performed using the Automated Meteorological Data Acquisition System (AMeDAS) of the JMA. Total data points of the number of log_10_ CFU of TAV in gall tissues or skins of grapevine were 415 (*n* = 415), obtained from a total of 5 grapevines sampled at 23 different times in vineyard A, 23 times in vineyard B, 11 times in vineyard C, 11 times in vineyard D, 12 times in vineyard E, and 3 times in vineyard F.

In general, Bayesian methods provide flexibility in modeling assumptions that allow the development of models that capture the complex nature of real-world data (Kawaguchi, 2022a; Kawaguchi and Nanaumi, 2022). In this study, the HBM was defined as:


(1) $$q_{i}=\alpha_{1}+\beta_{1}C_{i}+\beta_{2}L_{i}+\beta_{3}R_{i}+\beta_{4}T_{i}+\beta_{5}S_{i}$$

(2) $$x_{i}=\alpha_{2}+\beta_{6}G_{i}$$

(3) $$y_{i}∼\;Normal\;(q_{i}\sigma_{1}^{2})$$

(4) $$C_{i}∼\;Normal\;(x_{i}\sigma_{2}^{2})$$

(5) $$\sigma_{1}^{2}∼\;Uniform\;\left(0,\;1.0e+4\right)$$

(6) $$\begin{array}{l} \sigma_{2}^{2}\;∼\;Uniform\;\left(0,\;1.0e+4\right) \end{array}$$

where *q_i_* is the number of log_10_ CFU of TAV in gall tissues or skins of grapevine, *i* is the number of grapevine trees, *y_i_* is the posterior distribution of *q_i_* according to the normal distribution (mean = 0, variance = $\sigma_{1}^{2}$ ), $\sigma_{1}^{2}$ is given as a hyper-parameter and defined as a variable according to uniform distribution (mean = 0, variance = 1.0e+4) (non-informative prior distribution), and *x_i_* is the categorical variable of cultivars (categorical variable: 1, cv. Zweigeltrebe, 2, cv. Kerner; 3, cv. Auxerrois). Furthermore, *C_i_* is the posterior distribution of *x_i_* according to the normal distribution (mean = 0, variance = $\sigma_{2}^{2}$ , $\sigma_{2}^{2}$ is given as a hyper-parameter and defined as a variable according to uniform distribution (mean = 0, variance = 1.0e+4) (non-informative prior distribution), *α_1_ and α_2_* are the *y*-intercept as fixed effects, and *β_1_*, *β_2_*, *β_3_*, *β_4_*, *β_5_*, and *β_6_* are the coefficient variables associated with explanatory variables *C_i_* (cultivar, categorical variables described above), *L_i_* (locations, categorical variables: 1, Yoichi; 2, Urausu; 3, Furano; 4, Sapporo), *R_i_* (amount of rain per month (mm)), *T_i_* (average of temperature per month (°C)), *S_i_* (snow depth (cm)), and *G_i_* (Individual sampled-grapevine trees, categorical variables) as fixed effects, respectively. In equation 2, we assumed that the parameter *C_i_* (cultivar) is influenced by the variation in *G_i_* (individual grapevine trees) as a hyper-parameter. The objective variables are the log_10_ CFU counts of TAV in grapevine gall and skin tissues.”

Equations 1 and 2 are used to determine the posterior distribution of parameters based on the likelihood of the data and the choice of priors. To obtain unbiased samples of this posterior distribution, we used the Markov Chain Monte Carlo (MCMC) method in a fully Bayesian approach. In this study, we utilized the R package “cmdstanr” to estimate the coefficient variables through MCMC methods. RStan, the R interface to Stan, was used by cmdstanr. Stan utilizes the No-U-Turn Sampler (NUTS) (Hoffman and Gelman, 2014) to generate posterior simulations, given a user-specified model and data. We executed four independent calculation chains, each with 10,000 iterations, including a burn-in period of 1,000 iterations at the beginning of each MCMC run. We considered a value of the potential scale reduction factor Rê (R-hat) ≤ 1.05 as an indication of chain convergence (Gelman et al., 2015).

### Bayesian changepoint detection

All the data obtained in Yoichi and Sapporo were used because these locations had the highest number of population data and continuous months, including the snowfall season, compared to other locations where no data were collected from January to March.

In general, the Cauchy distribution is used as a prior distribution in the Bayesian changepoint detection (BCD) method (Peluso et al., 2019). The cumulative distribution function of Cauchy (*y*|*μ*, *σ^2^*) is defined as:


(7) $$F\left(y\right)\;=\;(1/\pi )\;arctan\;\{(y-\mu )/\sigma^{2})\}\;+\;0.5$$

Then, the inverse function of *F*(*y*) is defined as:


(8) $$F^{-1}\left(x\right)\;=\mu +\sigma^{2}tan\;\{\pi (x–\;0.5)\}$$

When *x* follows uniform distribution (average = 0, variance = 1), equation 8 follows the Cauchy distribution (average = *μ*, variance = *σ*). In this study, thus, the BCD was defined as:


(9) $$\mu_{t}=\mu_{t-1}+\sigma^{2}tan\;\{\pi (x_{t-1}–\;0.5)\}+\beta_{7}R_{t-1}+\beta_{8}T_{t-1}+\beta_{9}S_{t-1}+r$$

(10) $$x_{t-1}∼\;Uniform\;\left(0,\;1\right)$$

(11) $$Y_{t}∼\;Normal\;(\mu_{t},\sigma_{y}^{2})$$

(12) $$\;\sigma_{y}^{2}∼\;Uniform\;\left(0,\;1.0e+4\right)$$

(13) $$r∼\;Normal\;(0,\sigma_{r}^{2})$$

(14) $$\;\sigma_{r}^{2}∼\;Uniform\;\left(0,\;1.0e+4\right)$$

where *μ_t_* is the number of log_10_ CFU of TAV in gall tissues or skins of grapevine, *t* is the number of investigation-months, *x_t-1_* is defined as a variable according to uniform distribution (mean = 0, variance = 1), *Y_t_* is the posterior distribution of *μ_t_* according to the normal distribution (mean = 0, variance = $\sigma_{y}^{2}$ ), $\sigma_{y}^{2}$ is given as a hyper-parameter and defined as a variable according to uniform distribution (mean = 0, variance = 1.0e+4) (non-informative prior distribution), and *r* is the *y*-intercept as fixed effects defined as a variable with a normal distribution (mean = 0, variance = $\sigma_{r}^{2}$ ). Furthermore, $\sigma_{r}^{2}$ is given as a hyper- parameter and defined as a variable according to uniform distribution (mean = 0, variance = 1.0e + 4) (non-informative prior distribution), and *β_7_*, *β_8_*, and *β_9_* are the coefficient variables associated with explanatory variables, *R_t-1_* (amount of rain per month), *T_t-1_* (average of temperature per month), and *S_t-1_* (snow depth) as fixed effects as same as coefficients in equation 1, respectively. This analysis was carried out using the R package “cmdstanr” to estimate the coefficient variables through the MCMC methods described above.

### Population measurement of TAV in grapevine skins above and under the snow

To monitor TAV populations, sampling was conducted twice: first in March 2022 and then in January 2023 in a vineyard located in Yoichi. In 2022, five grapevines that were randomly selected were approximately 15 years old and of the *V. labrusca* × *V. vinifera* cv. Delaware variety and sampling was performed from four different ports per grapevine, resulting in a total of 20 samples (n=20). In 2023, three grapevines that were randomly selected were approximately 15 years old and of the *V. labrusca* cv. Campbell’s Early variety and sampling was performed from five different ports per grapevine, resulting in a total of 15 samples (*n*=15). Both the skin above the snow and the skin under the snow (approximately 1.0 g fresh weight per plant) were collected from the same grapevine. Isolation and enumeration of CFUs on Roy and Sasser medium were conducted using the same procedure as described above. The R software was used to perform a Student’s t-test.

## Results

### Population dynamics of TAV in gall tissues, grapevine skins, and snow in vineyards

**Figure 1:**
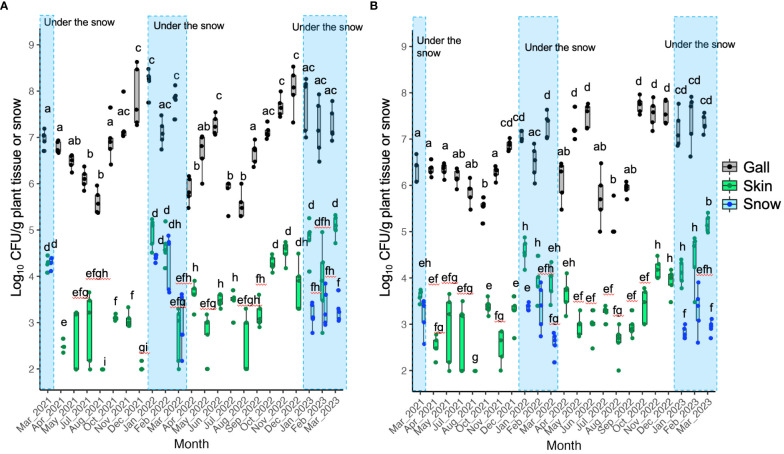
Population dynamics of pathogenic bacteria (tumorigenic *Allorhizobium vitis*) causing grapevine crown gall (GCG) in Yoichi: **(A)** Data from grapevine cv. Zweigeltrebe trees in vineyard A; **(B)** Data from grapevine cv. Kerner trees in vineyard **(B)** Pathogenic bacteria were isolated from galls, skins, and snow covering the skins (during the snowfall season only). The center bar of the box plot represents the median, while the lower and upper horizontal bars show the 25th and 75th percentiles respectively. The whiskers indicate the 95% range. Boxes labeled with different letters indicate a significant difference from the other boxes (*P* ≤ 0.05, Tukey’s HSD test).

**Figure 2:**
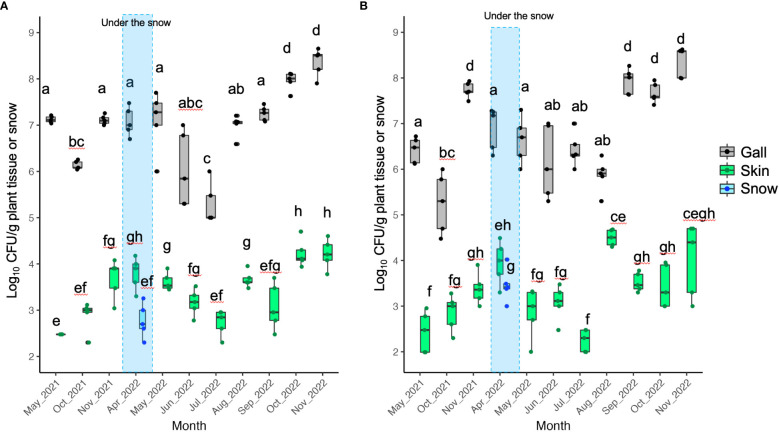
Population dynamics of pathogenic bacteria causing GCG in Urausu: **(A)** Data from grapevine cv. Zweigeltrebe trees in vineyard C; **(B)** Data from grapevine cv. Kerner trees in vineyard D Pathogenic bacteria were isolated from galls, skins, and snow covering the skins (during the snowfall season only). The center bar of the box plot represents the median, while the lower and upper horizontal bars show the 25th and 75th percentiles respectively. The whiskers indicate the 95% range. Boxes labeled with different letters indicate a significant difference from the other boxes (*P* ≤ 0.05, Tukey’s HSD test).

**Figure 3:**
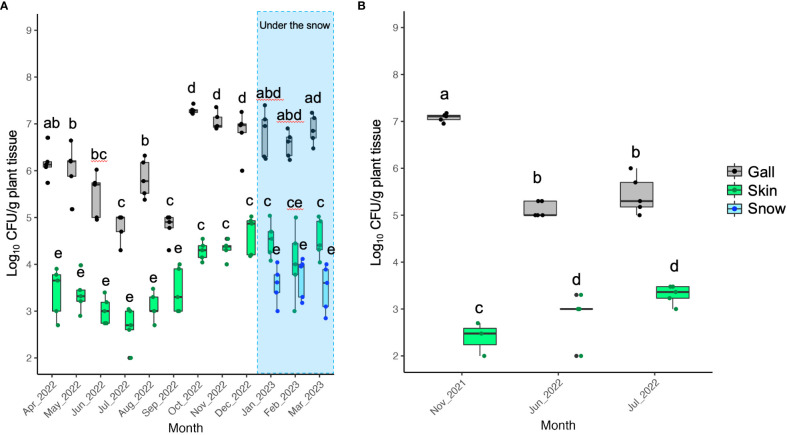
Population dynamics of pathogenic bacteria causing GCG: **(A)** Data from grapevine cv. Auxerrois trees in vineyard E in Sapporo; **(B)** Data from grapevine cv. Zweigeltrebe trees. in vineyard F in Furano. Pathogenic bacteria were isolated from galls, skins, and snow covering the skins (during the snowfall season only). The center bar of the box plot represents the median, while the lower and upper horizontal bars show the 25th and 75th percentiles respectively. The whiskers indicate the 95% range. Boxes labeled with different letters indicate a significant difference from the other boxes (*P* ≤ 0.05, Tukey’s HSD test).

The CFU of TAV was detected throughout the seasons from galls, skins, and snow samples obtained from infected grapevines. However, the CFU detected from gall tissues was significantly higher than that from skins and snow samples in the same month (**Figures 1**– **3**). Our results indicate that the snow covering infected grapevine skins was contaminated with TAV strains (**Figures 1**, **2**), while TAV strains were not isolated from the skins and the snow covering healthy grapevines (data not shown).

### Regression analysis by HBM model

The hierarchical Bayesian model (HBM) used log_10_ CFU of TAV in gall tissues as the objective variable and showed that the explanatory variables “cultivar” (mean = -0.323, Bayesian 95% credible interval (CI) = -0.459 to -0.187) and “temperature” (mean = -0.068, Bayesian 95% CI = -0.082 to -0.053) had significantly negative coefficients within the range of Bayesian 95% CI (**Table 1**). The negative coefficient for “cultivar” suggests that the difference in cultivars had a larger effect on the TAV population in galls. Similarly, the negative coefficient for “temperature” suggests that lower temperatures had a larger effect on the TAV population in galls.

**Table 1:** Results of coefficient estimation as posterior distribution as Bayesian modelling for factors related with population of Allorhizobium vitis (Ti) in gall tissues of grapevine in snowy vineyards by Markov Chain Monte Carlo (MCMC) method.

Objective variable	Coefficients	Explanatory variable	mean	Standard deviation (SD)	R-hat	Bayesian 95% credible interval	
						Lower	Upper
Logio CFU of A. vitis (Ti)/ggall tissse	*β*_1_	Cultivar	-0.323	0.069	1.000	-0.459	-0.187
	*β*_2_	Location	0.012	0.049	1.000	-0.085	0.107
	*β*_3_	Rain	0.000	0.001	1.000	-0.001	0.002
	*β*_4_	Temperture	-0.068	0.007	1.000	-0.082	-0.053
	*β*_5_	Snow depth	-0.002	0.001	1.000	-0.003	0.001
	*α*_1_	y-Intercept	7.515	0.165	1.000	7.192	7.837
	*σ* ^2^_1_	Variance of normal distribution	0.751	0.028	1.000	0.698	0.817
Cultivar	*β*_6_	Tree	0.046	0.003	1.000	0.040	0.051
	*α*_2_	y-Intercept	1.087	0.048	1.000	1.004	1.171
	*σ* ^2^_2_	Variance of normal distribution	0.515	0.019	1.000	0.480	0.553

In contrast, the model, using log_10_ CFU of TAV in skin tissues as the objective variable, showed that the explanatory variables “location” (mean = 0.153, Bayesian 95% CI = 0.055 to 0.252) and “temperature” (mean = -0.038, Bayesian 95% CI = -0.053 to -0.023) had significantly positive and negative coefficients within the range of Bayesian 95% CI, respectively (**Table 2**). The positive coefficient for “location” suggests that the difference in locations had a larger effect on the TAV population in skins. The negative coefficient for “temperature” suggests that lower temperatures had a larger effect on the TAV population in skins. All estimated coefficient values had an R-hat value of 1000, indicating that the MCMC runs successfully converged.

**Table 2:** Results of coefficient estimation as posterior distribution as Bayesian modelling for factors related with population of Allorhizobium vitis (Ti) in skin tissues of grapevine in snowy vineyards by Markov Chain Monte Carlo (MCMC) method.

Objective variable	Coefficients	Explanatory variable	mean	Standard deviation (SD)	R-hat	Bayesian 95% credible interval	
						Lower	Upper
Log10 CFU of A.	*β*_1_	Cultivar	0.033	0.071	1	-0.107	0.172
vitis (Ti)/g	*β*_2_	Location	0.153	0.05	1	0.055	0.252
skin tissse	*β*_3_	Rain	0.001	0.001	1	-0.001	0.002
	*β*_4_	Temperture	-0.038	0.008	1	-0.053	-0.023
	*β*_5_	Snow depth	0.002	0.001	1	-0.001	0.004
	*α*_1_	y-Intercept	2.632	0.183	1	2.273	2.994
	*σ* ^2^_1_	Variance of normal distribution	0.78	0.029	1	0.726	0.839
Cultivar	*β*_6_	Tree	0.05	0.003	1	0.044	0.055
	*α*_2_	y-Intercept	1.047	0.041	1	0.966	1.127
	*σ* ^2^_2_	Variance of normal distribution	0.491	0.018	1	0.458	0.528

### Trend of the population of TAV strains throughout the season analyzed by BCD

**Figure 4:**
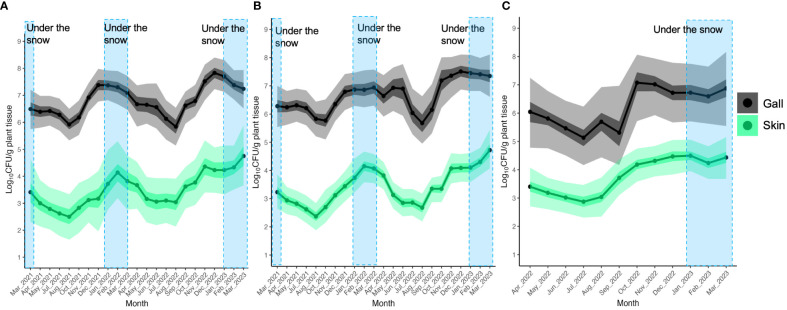
Population trend of pathogenic bacteria causing GCG throughout the season analyzed by Bayesian changepoint detection (BCD): **(A)** Data from grapevine cv. Zweigeltrebe trees in vineyard A in Yoichi; **(B)** Data from grapevine cv. Kerner trees in vineyard B in Yoichi; **(C)** Data from grapevine cv. Auxerrois trees in vineyard E in Sapporo. The dark shading represents the 50% Bayesian confidence interval, while the light shading indicates the 95% Bayesian credible interval.

In order to detect any trends in the dynamics of TAV CFU counts on/in gall and skin tissues over months or seasons, we used the BCD method with TAV population data from Yoichi and Sapporo. The results of the BCD analysis showed that in Yoichi, TAV populations isolated from both Zweigeltrebe and Kerner cultivars in gall and skin tissues increased from August to December 2021, gently decreased starting from April 2022, and especially dropped from June to August 2022, before increasing again from August to December (**Figures 4A, B**). These findings suggest that TAV populations increase in autumn (from September to December), remain high during winter (from January to March), decrease, and remain low during spring and summer in 2021 and 2022 (**Figures 4A, B**). In grapevines of the Auxerrois cultivar in Sapporo, TAV populations on/in gall tissues rapidly increased from September to October 2022, while populations on/in skin tissues gently increased from August to December 2022 (**Figure 4C**). These results from Sapporo are consistent with those obtained from Yoichi, suggesting an increase in TAV populations in autumn (from September to December).

### Population measurement of TAV in grapevine skins above and under the snow

**Figure 5:**
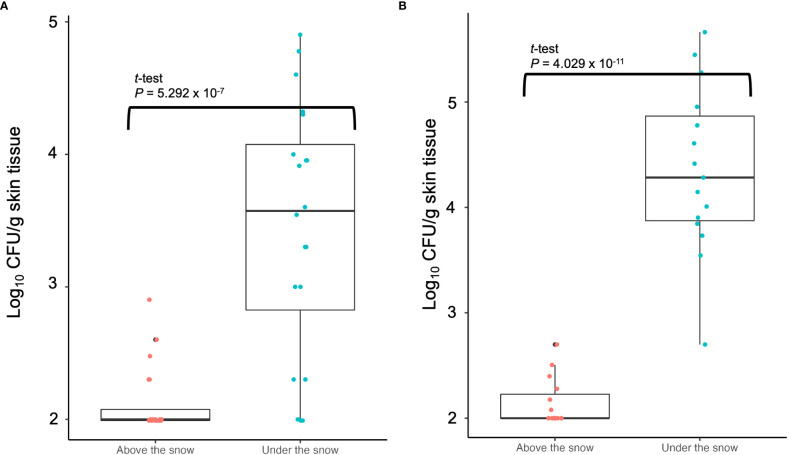
Population measurement of pathogenic bacteria causing GCG in grapevine skins above and below snow cover: **(A)** Data from grapevine cv. Delaware trees in 2022; **(B)** Data from grapevine cv. Campbell’s Early trees in 2023. The center bar of the box plot represents the median, while the lower and upper horizontal bars show the 25th and 75th percentiles respectively. The whiskers indicate the 95% range.

At the sampling sites of grapevine skins under the snow in 2022, 2.13 ± 0.26 (mean ± standard deviation of the mean) log_10_ CFU/g skin tissue was detected (**Figure 5A**). Furthermore, 3.45 ± 0.95 log_10_ CFU/g skin tissue was detected at the sampling sites above the snow (**Figure 5A**). The CFU/g skin tissue significantly differed (*P* ≤ 0.001) between the sites under and above the snow (**Figure 5A**). In the experiment in 2023 as independent replication, the result showed the same trend that the CFU/g skin tissue significantly differed (*P* ≤ 0.001) between the sites under and above the snow (**Figure 5B**).

## Discussion

Freezing injuries can cause wounds on/in vines and are considered a risk factor for promoting the occurrence of GCG (Burr et al., 1998; Kawaguchi et al., 2017; Kawaguchi et al., 2021). Therefore, GCG should not occur in snow-covered areas where freezing injuries are rare. However, outbreaks of GCG have been reported in Hokkaido, a region famous for heavy snowfall in Japan (Kawaguchi et al., 2021; Kawaguchi and Nanaumi, 2022). In this study, we focused on the population of TAV, a pathogenic bacterium causing GCG, in grapevine skins above and under the snow. Our results showed that the population of TAV under the snow was approximately 10-fold higher than that above the snow, suggesting that snow covering could maintain the number of TAV cells on the skins of grapevines. Although snow can prevent freezing injuries, it could also protect bacterial populations on grapevine skins due to stable low temperatures (approximately 0°C), moisture retention, and protection from air drying and UV radiation from the sun. Our results suggest that TAV under the snow could be protected and survive well during the snowfall season, leading to frequent occurrences of GCG in the snowfall regions of Japan. We also found that TAV survived in the snow attached to the skin surface of infected grapevines, indicating that melted snow in spring could become an inoculum for soil infection. Additionally, TAV survived in galls and skins throughout the seasons, suggesting that the pathogen might circulate from the soil to roots, inside plants, galls, skins, snow on skins, and falling to the ground through seasons. However, this idea needs to be verified using other investigative approaches in the near future.

Throughout this study, the TAV strains in skin tissues or snow samples were under 5.0 log_10_ CFU/g (≈ 10^5^ cells/g or mL). Although the incidence of gall formation was less than 15% in grapevine stems inoculated with 10^5^ cells/mL of TAV, it increased to over 80% when inoculated with 10^7^ cells/mL (Kawaguchi, 2014). Even low populations of TAV strains on/in skins and snow could infect grapevines, posing a potential threat if they spread around vineyards by melting snow, rainfall, or strong winds. Furthermore, cutting infected galls and skins might contaminate the scissors. Previous studies have suggested that the systematic survival of TAV strains in grapevines and soil, along with wounding from freezing injuries, is a key point in the disease cycle of GCG (Burr et al., 1998). However, there have been no in-depth epidemiological studies of GCG in snowfall regions, and our results provide the first insight into the population dynamics of TAV on/in galls, skins, and snow in vineyards throughout the seasons.

In this study, the results of the HBM analysis suggested that lower temperatures had a significant impact on the population of TAV on/in galls and skins, indicating that low temperature is a common factor affecting the TAV population in grapevines. Hokkaido, where the study was conducted, is known for its cold climate, with an average temperature in summer of approximately 23°C (JMA, http://www.jma.go.jp/jma/indexe.html). TAV can grow in temperatures up to 28°C, but growth is strongly inhibited at around and above 30°C (Kawaguchi et al., 2005b; Kawaguchi and Inoue, 2012). Even in Hokkaido, the number of days when the maximum temperature exceeds 30°C is increasing in summer. This could be one of the reasons for the decreasing TAV population during the summer. In addition, the study found that the TAV strains were able to survive in galls throughout the seasons, with a high population of TAV found under the snow during the winter, indicating that they did not die at low temperatures even during the winter and could be preserved in large numbers under the snow. However, Baron et al. (2001) have reported that the optimal temperature for TAV infection and virulence gene expression is approximately 20°C to 28°C and that the levels of many virulence proteins are significantly reduced at 28°C compared to 20°C. Thus, it appears that TAV strains can easily infect grapevines under the temperature conditions in summer, and GCG outbreaks are actually common in Hokkaido (Kawaguchi et al., 2021).

The BCD results showed that the population of TAV in galls and skins clearly changed from autumn and remained high during the snowfall season. This suggests that low temperatures and snowy conditions could facilitate the survival of TAV populations, which supports the results obtained from the HBM model regression analysis. Therefore, this study provides a new insight that the population dynamics of TAV on/in grapevines are season-dependent, which was previously unknown. Moreover, the trend of changing points in TAV population dynamics throughout the season was almost the same in the three different cultivars, Zweigeltrebe, Kerner, and Auxerrois, and the two different regions, Yoichi and Sapporo. Interestingly, the TAV populations in gall and skin tissues increased from August to December in 2021 and 2022, regardless of cultivar and location. In commercial vineyards, autumn fertilization is a common practice as it provides essential macronutrients such as nitrogen, phosphorus, and potassium to the plant at the right time to ensure adequate nutrition from the earliest stages. There is a possibility that the increasing TAV populations in gall and skin tissues could be related to plant nutrition conditions affected by autumn fertilization. Furthermore, grapevines typically grow and generate new shoots and leaves during spring and summer by utilizing stored nutrients from the previous autumn. Consequently, TAV populations may decrease due to reduced nutrient levels within the plants. Moreover, grapevines tend to become more active by high temperatures during the warmer months of spring and summer, and plant natural defense systems may be triggered, resulting in a further reduction of TAV populations. Furthermore, the plant defense systems may not be as effective in low temperatures during the colder seasons of autumn and winter, and it could allow TAV populations to increase.

Additionally, the microbial community present in grapevines with GCG symptoms may be linked to trends in the population dynamics of TAV (Gan et al., 2019). Generally, the microbial community in galls of grapevine is diverse and varies significantly across different samples and vineyards. Furthermore, the presence of TAV in the gall microbiota was consistently accompanied by *Xanthomonas* and *Novosphingobium* (Gan et al., 2019). However, the population dynamics of these organisms were not investigated throughout the seasons in the previous study. Therefore, to confirm the role of plant nutrition conditions and microbial communities, alternative investigative approaches should be pursued in the near future.

The objective variable, CFU of TAV in galls, showed that “cultivar” was a significant explanatory variable. There was a trend that the CFUs from the grapevine cv. Zweigeltrebe were relatively higher than those from other cultivars. Although some cultivars of grapevine belonging to *V. vinifera* are susceptible to TAV infection (Burr et al., 1998), susceptibility to GCG development of cv. Zweigeltrebe has not been reported. Further research is needed to verify whether Zweigeltrebe is more susceptible than other cultivars.

The results of the HBM also indicated that the “location” parameter affected the population dynamics of TAV on/in grapevine skins. Kawaguchi (2022b) showed that inferior growth due to gall formation could be caused by various factors depending on vineyard locations, such as excessive or insufficient water, rainfall, temperature, wind, storms, soil, and nutrition condition. Therefore, the difference in population dynamics of TAV on/in grapevine skins could be influenced by differences in the environment of each location. However, the regression analysis using the HBM showed that lower temperature was a common and important factor affecting the TAV population on/in both galls and skins.

In conclusion, this study aimed to elucidate the population dynamics of TAV in snowy areas using galls, skins, and snow samples obtained from infected grapevines across four locations in Hokkaido, Japan. The findings revealed that TAV can survive well under snow during the snowfall season, and can overwinter in galls and on/in skins under the snow, potentially serving as a pathogen for the next season. While microbial diversity in grapevines has been explored in various countries (Gan et al., 2019), little is known about its diversity in snowy regions. The technical and biological insights gained from this study provide a better understanding of the complex interactions between grapevines and their microbial communities in snowfall regions, which could facilitate better management of GCG in the future. Moreover, the antagonistic AV strains ARK-1 and VAR03-1, previously reported (Kawaguchi et al., 2008; Kawaguchi et al., 2017; Kawaguchi, 2022c), could aid in controlling GCG in commercial vineyards located in snowfall regions.

## Data availability statement

The raw data supporting the conclusions of this article will be made available by the authors, without undue reservation.

## Author contributions

All authors consent to this submission. Author Contributions: Conceptualization, AK and YM; methodology, AK; investigation, AK, SO, YM, TSa and TSo; formal analysis, AK; writing, AK; validation, AK; supervision, AK; data curation, AK; resources, AK, TSa, and TSo.
